# Is the Laminar Flow From the Vitrectomy Infusion Cannula Potentially Harmful?

**DOI:** 10.1167/tvst.14.11.29

**Published:** 2025-11-19

**Authors:** Tommaso Rossi, Giorgio Querzoli, Pier Giuseppe Ledda, Bjorn O. Balzamino, Camilla Pellizzaro, Giulia Rosari, David H. Steel, Rodolfo Mastropasqua, Mario R. Romano, Alessandra Micera

**Affiliations:** 1 IRCCS - Fondazione Bietti ONLUS – Roma, Italy; 2 DICAAR – Università degli Studi di Cagliari, Cagliari, Italy; 3 Department of Biomedical Science, Humanitas University, Milan, Italy; 4 University of Chieti – Department of Ophthalmology, Chieti, Italy; 5 Biosciences Institute, Newcastle University, Newcastle Upon Tyne, UK

**Keywords:** pars plana vitrectomy, infusion cannula, flow rate, computational fluid dynamics, RNA transcriptomics

## Abstract

**Purpose:**

To model the jet flow of a 25G vitrectomy infusion cannula onto the opposite retina in a balanced salt solution–filled eye, calculate pressure and shear stress at the retina, and evaluate the biologic response to the localized increase of pressure on an ex vivo pig retina model.

**Methods:**

Fluidics, pressure, and shear stress were calculated using computational fluid dynamics. Ten pig eyes within 4 hours of slaughter were prepared to receive a 10 mL/min flow for 1 or 3 minutes after removing the vitreous and inserting a 25G infusion cannula in the usual fashion. RNA transcripts of selected proteins involved in inflammation, oxidative stress, and apoptosis were amplified and measured.

**Results:**

At a 10 mL/min infusion flow rate, the average fluid velocity at outlet reached 1.33 m/s and pressure at impact site increased more than 2 mm Hg in a region approximately 1 mm wide. RNA transcripts of p65, nuclear factor κB, casp3, p53, inducible nitric oxide synthase, Keap1, Nrf2, and glial fibrillary acidic protein reached statistical significance on analysis of variance at the 0.01 level and interleukin-1b and NOX4 exceeded 2log_2_ units fold-change.

**Conclusions:**

The jet flow generated by 25G vitrectomy infusion cannulas not only may compromise the surgeons’ view blowing on dyes and create potentially harmful perfluorocarbon liquid bubbles, it also reaches significant velocity and remarkable pressure at the impact site. RNA transcripts of proteins involved in inflammation, oxidative stress, and apoptosis significantly increase as soon as 1 and 3 minutes after flow starts.

**Translational Relevance:**

Evaluation of the infusion jet consequences on the retina lays the foundations for finding solutions that reduce pressure increase and tissue response.

## Introduction

Pars plana vitrectomy (PPV) is the elective surgical procedure for many vitreoretinal diseases including retinal detachment, proliferative diabetic retinopathy, epiretinal membranes, macular holes, and vitreous hemorrhage. The invariance of ocular volume and pressure during vitrectomy is essential to prevent severe complications and is accomplished by injecting balanced salt solution (BSS) through the infusion cannula at the flow rate required by aspiration.^1^

The introduction of dual cutting blades in 2014^2^ more than tripled the aspiration flow rate of small gauge (namely, 25G and 27G) vitrectomy probes,^3^^,^^4^ even at high cut rates, thus requiring a much higher infusion flow^5^ to maintain stable intraocular pressure (IOP) during vitrectomy. Flowrate results from the product of fluid velocity and the area of the internal lumen of the cannula: Q = U × A. Therefore, increasing the flow rate Q through small-bore infusion cannulas necessarily speeds up fluid, which, in turn, may imply safety issues.

The undesired effects of infusion cannulas laminar flow include the annoying dispersion of vital dyes and triamcinolone crystals layered onto the retinal surface, the formation of perfluorocarbon liquid^6^ bubbles potentially migrating into the subretinal space, and iatrogenic retinal breaks,^7^ yet uncommon, caused by the sudden opening of air or fluid infusion.^8–^^10^

The purpose of present study was to characterize the laminar infusion flow during 25G PPV in a BSS-filled eye, thus simulating the surgical condition typical of anterior vitreous shaving and vital dye removal, when posterior and core vitrectomy has been performed and the infusion jet stream reaches the retinal surface undisturbed. We used a combined approach comprising computational fluid dynamics to calculate BSS pressure at the retinal surface, and RNA transcriptomics restricted to selected target sensors for retinal damage, to evaluate the retinal response to infusion flow barometric insult on an ex vivo pig eye model.

## Materials and Methods

### Computational Fluid Dynamics

**Figure 1.**
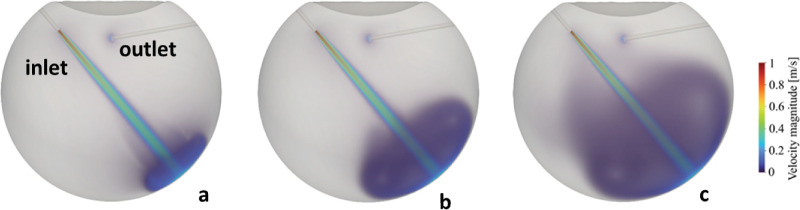
Development of the infusion jet at 10.0 mL/min flow rate. (**a**) At 0.15 seconds after the start of the infusion. (**b**) At 0.75 second after the start of the infusion. (**c**) At 4.0 seconds after the start of the infusion. Colors represent the velocity magnitude in meters per second. (**a**) Inlet (infusion cannula) and outlet (vitrectomy probe tip) sections are indicated in *red*. The vitreous chamber diameter is 24 mm.

Unsteady direct computational fluid dynamics simulations of the BSS infusion were performed using the OpenFOAM environment. The model resolves the mass conservation and Navier–Stokes equations for an incompressible Newtonian fluid with dynamic viscosity μ = 10^−3^ Pa·s and density ρ = 997 kg/m^3^. The vitreous chamber mimics a typical eye geometry and is the same as in previous studies:^11^ a 24-mm sphere with a convex anterior indentation. Two circular cylinders, representing 25G needles (external diameter 0.5 mm, internal diameter 0.4 mm), are positioned based on typical surgical guidelines. The infusion cannula enters perpendicularly to the eye through the pars plana (40° from the center of the eye) and penetrates the vitreous chamber for 3 mm. The aspiration cannula enters with the same angle, but the outlet surface is located 3 mm below the crystalline lens, as shown in Figure 1a. The flow inside the vitrectomy probe and infusion cannula is not computed, although they are geometrically represented in the computational domain and boundary conditions are imposed at the tips (*red sections* in Fig. 1a). At the inlet, a parabolic velocity profile was imposed with magnitudes modulated to obtain the prescribed flow rate. We considered seven flow rates ranging from 7.5 to 20 mL/min, each of them assumed constant during infusion, whereas at the outlet we imposed a constant pressure tuned to obtain an IOP (defined as the average pressure within the vitreous chamber) as high as 16 mm Hg. We simulated the time evolution of the phenomenon until the transient effects disappeared, that is, to a maximum time of 25 and 10 seconds for the lowest and highest flow rates, respectively. The fluid mechanics equations were solved on an unstructured mesh comprising approximately 2,000,000 hexahedral and tetrahedral cells, which was generated using the “snappyHexMesh” tool available in the OpenFOAM library. To accurately model the interactions in the eye surface neighborhood, three layers of hexahedral cells of progressively increasing height were extruded from the eye surface. A no-slip boundary condition was applied for velocity at the walls. As concerns the numerical discretization, we exploit second-order spatial schemes. Time marching is obtained through a second order backward differentiation formula. A mesh convergence analysis on the highest velocity case revealed a discretization error of less than 1% as the mesh is refined, in terms of average velocity within the vitreous chamber. Data were processed using ParaView 5.9.1 and MATLAB.

### Experimental Setting

This study was approved by the Institutional Review Board of the IRCCS Fondazione Bietti and all experiments were conducted following the Association for Research in Ophthalmology Statement for the Use of Animals in Ophthalmic and Vision Research.

To replicate the effects of infusion cannula laminar flow on the retina, we used 10 pig eyes within 4 hours of slaughter, excised the anterior segment (cornea and lens), gently removed the vitreous gel and filled the chamber with balanced saline solution and then exposed one retinal quadrant to a 10 mL/min flow rate through a 25G infusion cannula (Optikon 2000, BVI, Waltham, MA) inserted into its own trocar cannula and held at an 18-mm distance from the retina, to replicate the regular surgical placement. The infusion cannula was connected to a Balanced Salt Solution bottle at 22°C (BSS; BVI). The infusion bottle was placed 0.58 m above the pig eye, obtaining 10 mL/min flow rate, as measured by flowmeter (SonoFlow IL.52/3 V2. Sonotec; Hauppauge, NY) along the line.

The pig eyes were exposed to the infusion jet for a duration of 60 or 180 seconds (hereafter indicated with T1 and T3). Then the pig retina was immediately dissected into quadrants and fixed, marking the quadrant hit by the laminar flow and using the remainder retina as control. The experiment was repeated five times on different eyes for each duration. T0 indicates the baseline condition after infusion cannula insertion and before starting any flow.

### RNA Extraction, cDNA Synthesis, and Transcript Amplification

The transcript expression of selected retinal proteins related to the clusters of inflammation, apoptosis, retinal activity and oxidative stress shown in Table 1, at T0, T1, and T3 retinal irrigation durations has been carried out by relative real-time polymerase chain reaction (PCR). Briefly, the involved and uninvolved (control) regions of retina were quickly collected at T0, T1, and T3 and extracted in Trizol and RNA was dissolved in 11 µL RNAse-free water (depc-treated and autoclaved MilliQ water), according to a standard procedure. A routine spectrophotometric analysis (1.5 µL total RNA per sample) was carried out for RNA quantification/assessment of quality (Nanodrop N1000). Retrotranscription (100 ng total RNA) was carried out in a LifePro Thermal Cycler using the reverse transcription kit in the presence of supplements (dNTPs, Oligo-dT, and RNasin) and thereafter cDNAs (3 µL/target and 1 µL/referring gene) were amplified using the Hot start SYBR green PCR Master Mix in a Biorad CFX96 Real-Time PCR System (Bio-Rad, Hercules, CA). All molecular-based reagents, including master mix, supplements, and molecular grade water, were from Fisher Molecular Biology (Rome, Italy). Experimental and control amplifications were performed in parallel. Cq values (Bio-Rad) from normalized samples showing one melting curve were run in a REST program. The 2^−ΔΔCt^ method was used to calculate differences (fold changes) in the expression of inducible nitric oxide synthase (iNOS) gene between T0, T1, and T3, and data were provided as log2 expression ratio relative to control retina, as described elsewhere in this article, and considering the H3 house-keeping gene. Primers were designed from GenBank mRNA complete sequences and using the Primer3 software and synthesized by Eurofin Genomics (https://eurofinsgenomics.eu/).

**Table 1.** RNA Transcripts Selectively Searched for at Baseline and 1 and 3 Minutes of Irrigation (T0, T1, and T3)

Role In	Protein Name and Abbreviation
Inflammation	p65, endonuclease (EDN), interleukin 1b (IL-1b)
Apoptosis	Caspase 3 (CASP3), p21, p53
Retinal activity	Glial fibrillary acidic protein (GFAP), inducible nitric oxide synthase (iNOS)
Oxidative stress	NADPH oxidase 4 (NOX4), nuclear factor erythroid 2 (NFR2), Kelch-like ECH-associated protein 1 (KEAP1)

### Statistical Analysis of Molecular Data

Molecular data have been reported mean ± standard deviation and statistical analysis used JASP 0.19.3 (Amsterdam, the Netherlands). Subgrouping included baseline (T0; before irrigation started), after 1 and 3 minutes (T1 and T3). The Shapiro–Wilk tests validated the Gaussian distribution assumption; REST-analysis of variance (ANOVA) coupled analysis, and ANOVA followed by pairwise comparisons with Tukey's test, were carried out to identify significant changes in real-time PCR experiments. RNA fold-change data were represented as log_2_, for relative real-time PCR. The 2 log_2_ units were considered biologically significant.

## Results

### Computational Fluid Dynamics

**Figure 2.**
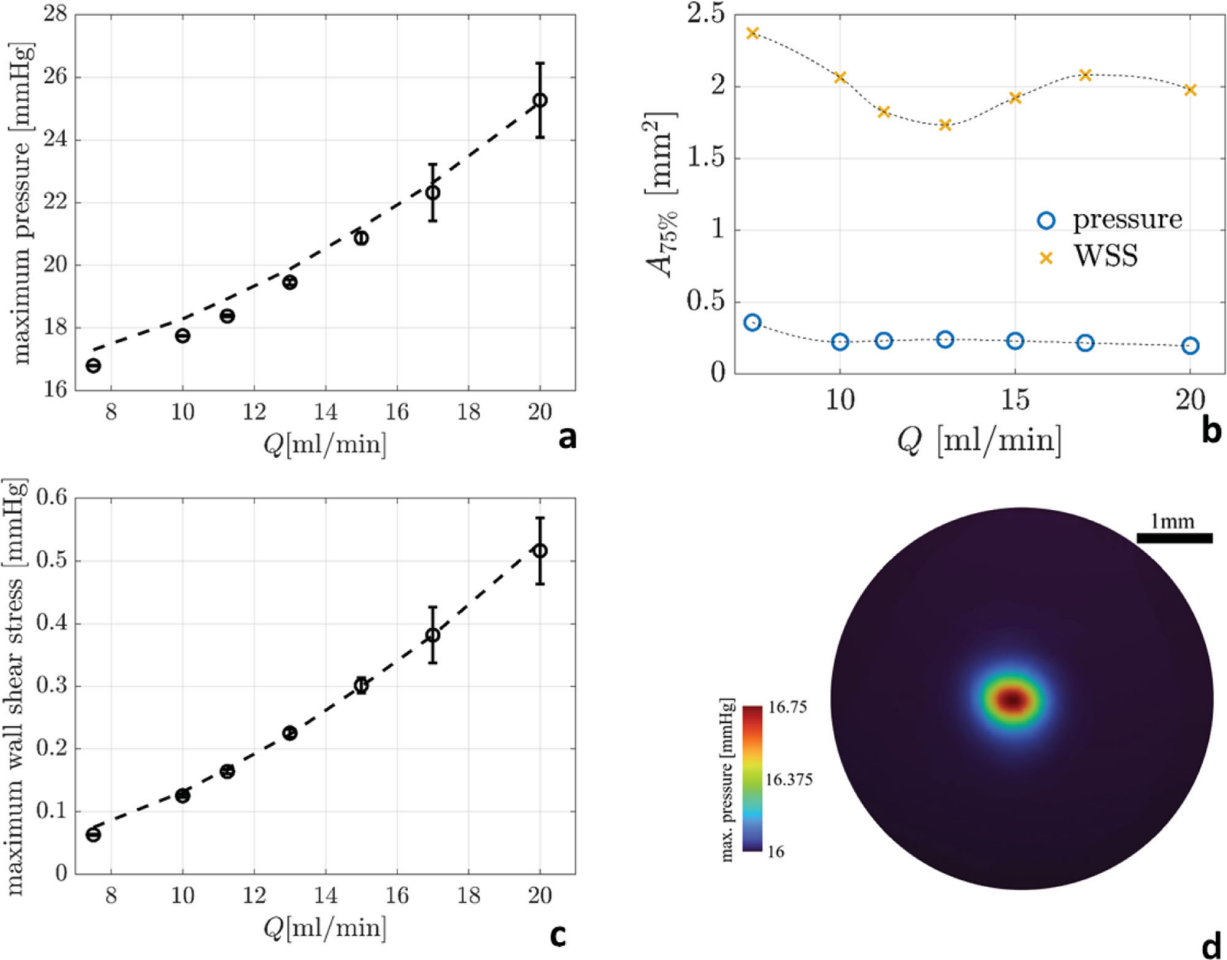
(**a**) Maximum time-averaged pressure, p_max_ (*circles*) ±1 standard deviation (*error bars*), at the impact point as a function of the flow rate. (**b**) Surface area (mm^2^) where the maximum jet-induced pressure, p_max_ – IOP (*circles*) and wall shear stress (*crosses*) exceed 75% of their maximum values at the given flow rate. The dashed line is a quadratic best fit of the data. (**c**) Maximum wall shear-stress (*circles*) ±1 standard deviation (*error bars*), around the impact point as a function of the flow rate. The dashed line is a quadratic best fit of the data. (**d**) Colormap of the maximum pointwise pressure observed during the jet development on the retinal surface around the impact point (mm Hg) at a 10.0 mL/min infusion flow rate. Color scale is intentionally set with a reduced maximum pressure value to enhance the map readability. WSS, wall shear stress.

The BSS exits the infusion cannula at 1.33 m/s for 10 mL/min flow rate and 2.65 m/s at 20 mL/min (Re = 529 at 10 mL/min and Re = 1058 at 20 mL/min) generating a laminar jet which cross the vitreous chamber hitting the opposite retina (Fig. 1). The maximum pressure (averaged in time after the transient) increases with the flow rate as shown in Figure 2a. Remaining laminar, the jet maintains its coherence and, at the point of impact, it is still compact; thus, the area where the pressure exceeds 75% of the maximum pressure is less than 0.5 mm^2^, irrespective of the flow rate.

The excess pressure relative to IOP ranges from 1 mm Hg at 7.5 mL/min to 9 mm Hg at 20 mL/min, with an approximately quadratic trend with the flow rate (*dashed line* in Fig. 2a).The wall shear stress is much lower, remaining at less than 0.5 mm Hg even at a flow rate of 20 mL/min (Fig. 1c). The increased standard deviations shown in Figure 1a and 1c indicate the onset of flow instability during development of the jet at the higher flow rates.

### Molecular Analysis Display Changes in Transcript Expression

**Figure 3.**
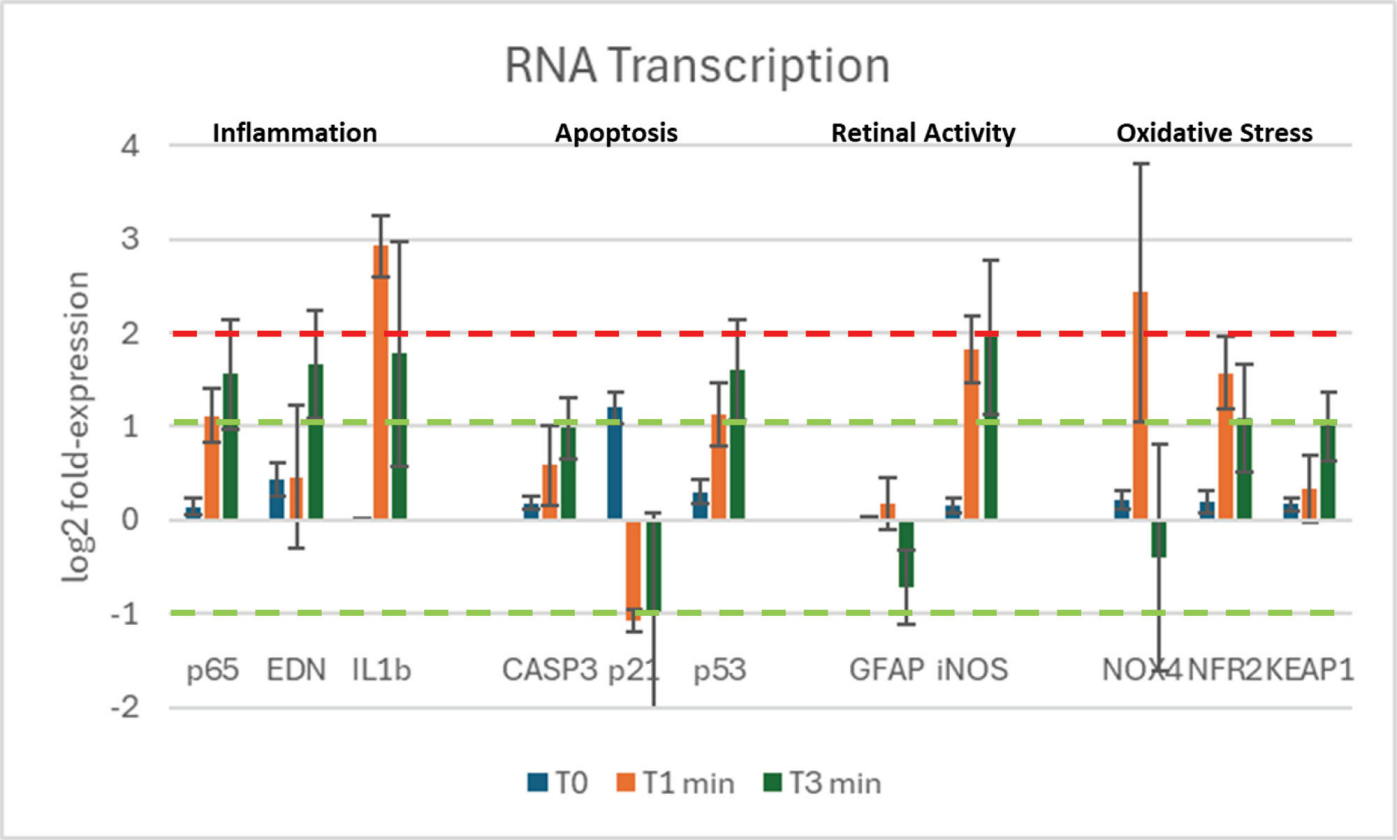
RNA transcription of selected target genes at T0, T1, and T3 (0, 1, 3 minutes) of BSS irrigation. Graphic of relative transcripts’ expression specific for p65, EDN, IL1β, CASP3, p21, p53, GFAP, iNOS, NOX4, and NRF2 NS Keap1, as grouped in clusters. Data are the result of amplifications from retinal extracts at T0, T1, and T3. The *dashed green line* represents the limit for statistical significance, and the *red line* refers to 2log-FC PCR high biological relevance cut-off.

**Figure 4.**
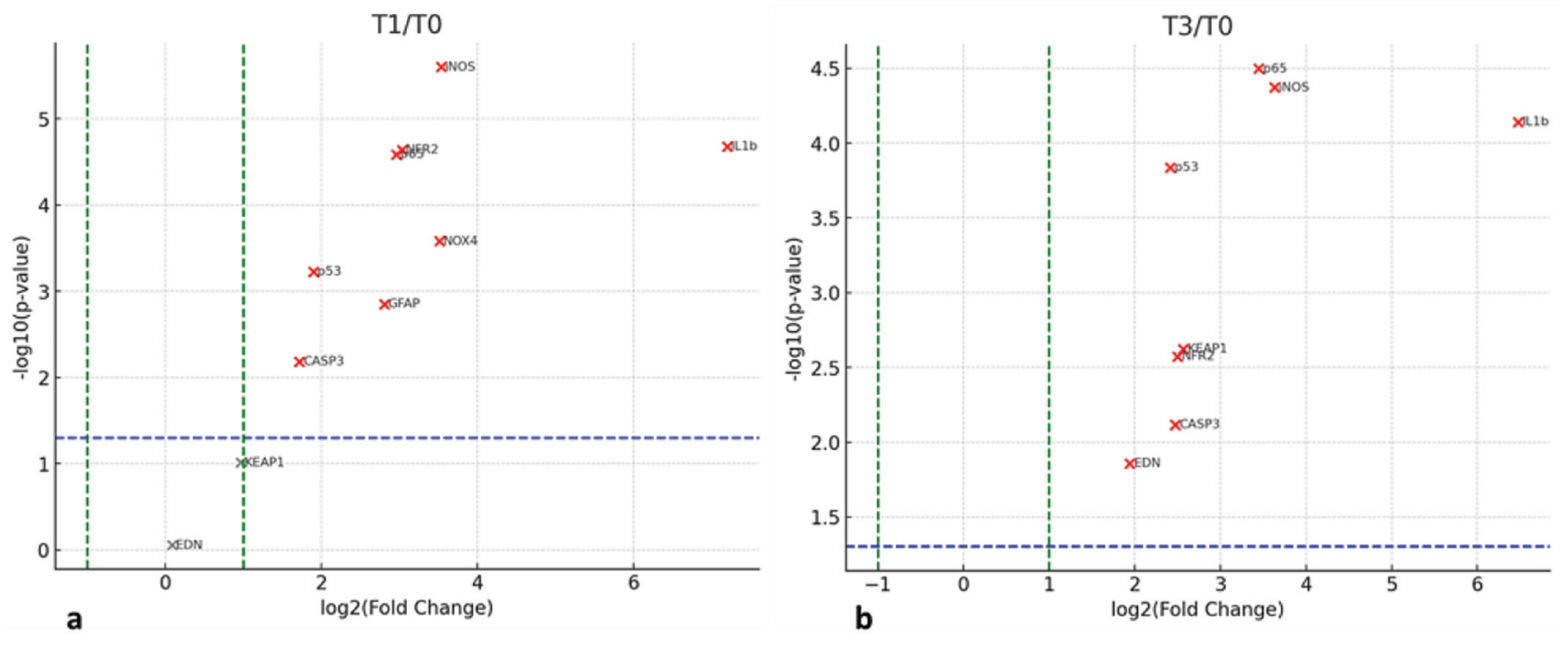
Volcano plot of RNA fold change as a function of statistical significance. (**a**) T1 versus T0 and (**b**) T3 versus T0. The *green dashed lines* mark −1and +1 LOG2 units and the *dashed blue horizontal line* marks the statistical significance (*P* = 0.05).

The transcript expression of selected targets reported in Figure 3 as log_2_ fold-change expression at baseline (T0), 1 minute (T1), and 3 minutes (T3) after starting irrigation. The relative RNA amplification of the retinal section directly hit by the laminar flow, compared to the opposite retina (control), was greater than 2 log_2_ units for IL1β and NOX4. ANOVA of fold-change data is also reported in Table 2. Many targets, including p65, nuclear factor κB, casp3, p53, iNOS, Keap1, and Nrf2, reached statistical significance at the 0.01 level, suggesting an upregulation at T1 that inverted at T3. Downregulation of p21, glial fibrillary acidic protein (GFAP), and NOX4 at T3 indicated an inverted expression pattern. Interestingly, downregulation was also observed for p21 as soon as at T1 (Figs. 3 and 4).

**Table 2.** ANOVA Results of log2 Fold-Change for All Selected Transcripts

	Clusters of Analysis										
	Inflammation			Apoptosis			Retinal Activity		Oxidative Stress		
Groups	p65NFkB	EDN	IL1β	CASP3	p21	p53	GFAP	iNOS	NOX4	NFR2	KEAP1
T0 Vs T1	<0.01	n.s.	<0.01	<0.01	<0.01	<0.01	<0.01	<0.01	<0.01	<0.01	n.s.
T0 Vs T3	<0.01	<0.01	<0.01	<0.10	<0.01	<0.01	<0.01	<0.01	n.s.	<0.01	<0.01
T2 Vs T3	<0.05	<0.01	**<0.001**	n.s.	n.s.	<0.05	<0.01	n.s.	**<0.001**	<0.05	<0.01

n.s., not significant.

See Table 1 for target gene abbreviations.

The bolded value was used to highlight statistical significance.

## Discussion

Matching aspiration and infusion flow rates during PPV maintains ocular homeostasis and prevents dangerous complications related to sudden hypotony^12^ or increased IOP.^13^ The introduction of dual blade cutters^14^ and the miniaturization of instruments raised the complexity of vitrectomy fluidics,^15^ demanding higher infusion flow rates to keep up with improved aspiration.^16^ Given the section restraints imposed by the gauge, the only way to increase infusion flow rate is to increase the velocity of the BSS entering the vitreous chamber through the cannula.

We calculated that the infusion cannula produces laminar flow during PPV: at a 10 mL/min flow rate, the average fluid velocity, which is the ratio of the flow rate to the lumen of the cannula, is as high as 1.33 m/s at the inlet. The infusion jet generates a very localized increase in pressure at the retinal impact site (2 mm Hg, corresponding with 266 Pa). The surgical relevance is debatable, however multifaceted, and goes from the annoying blowing on retinal vital dyes or triamcinolone crystals, owing to the high velocity of the entering fluid, that temporarily obscures the surgeon's view with a tinted or whitish cloud, to the formation of small perfluorocarbon bubbles that may migrate into the subretinal space. Jet flow–induced retinal tears^10^ have also been described, albeit rarely.

IOP variation during PPV is well-known and its magnitude may even exceed values shown Figure 2a. However, the delta of pressure determined by infusion laminar flow is extremely localized, whereas intraoperative IOP changes exert the same force throughout the entire retinal surface. Conceivably, perfusion control and compensating mechanism^17^ deployed when the entire retina is stimulated, can hardly be invoked at the very localized level, such as the 0.5 mm wide area hit by the jet stream. The presence of a biologic response witnessed by RNA transcriptome alteration represents another indirect evidence. Hasumura et al.^18^ conducted electron microscopy studies on rabbit retina hit by the air infusion laminar flow and demonstrated that the presence of clear-cut retinal damage circumscribed to the site of retinal impact, no larger than 0.5 mm in diameter, where the inner limiting membrane detached with exposure of Muller cells end plates and retinal nerve fibre layer bundles. Interestingly, the reported size of damage matches our results shown in Figure 2b.

The ex vivo pig eye model demonstrates that the laminar flow barometric insult clearly evokes a biologically measurable response with a statistically significant increase of most tested proteins RNA transcription (Table 2 and Fig. 3) and a biologically very relevant raise in interleukin-1b (IL-1b) and NOX4. Inflammation proteins (p53, EDN,^19^ and IL-1b^20^) increased (Fig. 3; Table 2), as well as oxidative stress related proteins (NOX4, NFR2, and KEAP1); apoptosis markers also changed with CASP3 and p53 increasing, whereas p21 was downregulated (Fig. 3). An interesting point is related to the expression of GFAP and iNOS, well-known sensitive biomarkers of neuroglia activation, mainly Muller cells and retinal neurons.^21^ Both GFAP^22^ and iNOS expression can change dramatically in response to a variety of stimuli, including mechanical^23^ stress of the retina. Our data indicate that GFAP is not involved, whereas iNOS, the source of NO, seems to respond to flow, probably by retinal ganglion cells that are the cells at the borderline of the vitreoretinal interface. Finally, with respect to KEAP1–NRF2 pathway, the principal protective response to oxidative, electrophilic stresses and cellular defense, we observed an inverted expression between Keap1/Nrf2 at T1 and a stabilization at T3 in concomitance with nox4 activation. Because Nox4 is a regulator of nuclear traffic of Nrf2, we can suppose the activation of mechanism of resilience by retinal cells, as observed in other tissues.

Although RNA transcription does not imply damage per se and many of coded proteins are also involved in repair mechanisms, our transcript data support the proof of concept that the retina senses the infusion laminar flow and reacts to it: the biologic pathway changes we measured clearly indicate a biological response and an intense distress signaling involving inflammation, apoptosis, and/or repair metabolisms.^24^^,^^25^

Interestingly, the geometry of PPV infusion cannulas, consisting of a mere hollow cylinder, has not received much attention in the past 50 years and remained unchanged, as if the infusion-related high-speed laminar flow hitting the retina were neglected or considered trivial. Indeed, very few modified infusion cannulas have been designed in the past and Hirata et al.^26^ demonstrated visual field defects consistent with air infusion cannula placement in 18% of 100 PPV for macular hole, suggesting the defect was to be ascribed both to dehydration of the retina and direct infusion jet trauma. They developed an infusion cannula with a closed bottom and four round openings, 90° apart^27^ and intended to prevent retinal drying during lengthy air infusion,^28^ before the introduction of valved trocars.

Other variants include Liu et al.’s design,^29^ which focused on bending the tubing insertion to minimize the risk of inadvertent dislocation but left the intraocular tract and the resulting laminar flow unmodified. Rickels et al.^30^ made 2D models of four different designs intended to reduce fluid velocity but never went any further.

In summary, we studied the fluid dynamics of vitrectomy infusion flow at rates routinely achieved during PPV and calculated pressure at the retinal surface; we also demonstrated that in addition to annoying blowing on vital dyes and perfluorocarbon bubble formation, the laminar flow induces a measurable biologic response possibly involving inflammation, oxidative stress and apoptosis.

The clinical and translational significance of the biologic response seen in the ex vivo pig model is debatable: the significant and very localized pressure increase could possibly determine a minute scotoma of less than 2° width in the midperiphery, depending on where the cannula points, which would be hardly noticed by the patient and easily missed by current visual field testing strategies with 4° grid spacing within 30° central degrees. We nonetheless believe that is important to establish whether jet stream can damage the retina and, in any case, a better cannula design reducing the blowing effect would be desirable.

Our study presents with limitations inherent to its design: the relatively low number of tested pig eyes and the selection of tested RNA transcripts can be widened and improved, as well as the design of an in vivo animal model to better represent the human surgical setting.

After more than 50 years, it may be time to redesign infusion cannulas, according to the modified fluidic dynamics of small gauge surgery and reduce velocity without compromising on flow rate. Inducing turbulence at the infusion outlet could represent a viable solution, although efficient design capable of abating fluid velocity before it reaches the retina remains to be deployed.
